# Maternal Kangaroo care education program in the neonatal intensive care unit improved mothers' perceptions, knowledge, perceived barriers and stress relates to premature infant

**DOI:** 10.1002/nop2.1311

**Published:** 2022-08-11

**Authors:** Sharmiza Samsudin, Ping Lei Chui, Azanna Binti Ahmad Kamar, Khatijah Lim Abdullah

**Affiliations:** ^1^ Department of Nursing Science, Faculty of Medicine Universiti Malaya Kuala Lumpur Malaysia; ^2^ Department of Pediatrics, Faculty of Medicine Universiti Malaya Kuala Lumpur Malaysia; ^3^ Department of Nursing School of Healthcare and Medical Sciences Sunway University Petaling Jaya Malaysia

**Keywords:** educational program, kangaroo care, knowledge, neonatal intensive care unit, perceived barriers, perceptions, premature infant, stress

## Abstract

**Aim:**

To assess the effectiveness of the maternal kangaroo care education programme over 1 month and 3 months on the mother's perception, knowledge, perceived barriers and stress.

**Design:**

A quasi‐experimental and longitudinal study was conducted among mothers with premature infants.

**Methods:**

Forty‐eight mother‐infant dyads were enrolled per arm in the control and experimental groups. The control group received standard routine care, while the experimental group received a maternal kangaroo care education program. Data were collected through self‐administered Kangaroo Care Questionnaires. Chi‐square, the general linear model and repeated measures ANOVA were used to analyse data.

**Results:**

The demographics are a majority of Malay mothers with multipara, a caesarean delivery with prematurity. At 3 months post‐intervention, the experimental group reported a significant reduction in stress, a positive perception and good knowledge towards kangaroo care implementation. The mothers' perceived barriers towards kangaroo care significantly decreased after 3 months in the experimental group.

## 1 INTRODUCTION

The World Health Organization (WHO) has estimated that 15 million babies are born prematurely worldwide every year, and the number is increasing (WHO, 2018). These preterm infants are nursed in incubators to maintain their temperature and to enable them to gain weight adequately. Whilst being nursed in incubators, the infants are separated from their mothers. Kangaroo care (KC) is a humanistic approach that began in Bogota, Colombia in the late 1970s as an alternative intervention in the neonatal intensive care unit (NICU) to maintain premature infants' body temperature when incubators were insufficient (Nieder‐Heitmann, 2010). It is based on a family‐centered care model which was developed to foster the role of mothers in the NICU (Davy et al., 2011). Kangaroo care refers to a period of care fostering bonding, attachment and interaction between mother and premature infant via skin‐to‐skin contact. The infant, who is naked except for a diaper is placed in an upright position against a parent's exposed chest with a piece of cloth covering his or her back. The concept behind this skin‐to‐skin contact between infant and parent is to enable the continued provision of warmth and love to the infant which is vital for the infant's growth (Nyqvist et al., 2010).

## 2 BACKGROUND

The uniqueness of KC has been accepted worldwide due to its enormous benefits in creating a balance of physiological indices while improving psychological status. It has been proven that KC helps to regulate body temperature, accelerate weight gain, increase sleep hours, improve oxygen saturation levels, reduce apnoea and decrease the infant's length of hospitalization. It significantly contributes to the reduction of overall infant morbidity and mortality (Ludington‐Hoe, 2013). Kangaroo care encourages infant‐mother connection, builds the mother's self‐confidence and enhances the breastfeeding period, which results in an increase in milk production that has an impact on the infant's response to pain and future behavioural problems (Blomqvist et al., 2012; Boundy et al., 2016; Davy et al., 2011; Ludington‐Hoe & Morgan, 2013; Verklan & Walden, 2015). Besides, KC assists in easing overcrowded nurseries, lessens the burden on scarce and costly resources such as incubators and reduces neonatal infection and infant mortality rates (Zaidi et al., 2005).

Kangaroo care has been established as a safe and effective method of premature infant care in the past three decades, with the potential to improve the survival of low birth weight infants, especially in low and middle‐income countries (WHO, 2019). In many countries in the South East Asia region including Malaysia, KC has not been implemented in a practical nor institutionalized manner. Instead, it is often used based on the instinct of the healthcare worker or upon advice from the attending consultant of the respective NICU. In other words, the practice may be carried out in tertiary hospitals, but there is no definitive protocol, guideline, or prior training to support it. There is also a dearth of literature surrounding this practice in NICUs in Malaysia and South East Asia. Information on KC may have been given verbally without clear instructions on the implementation of the proper technique.

Thus, the present study focuses on a specialized KC education programme that was designed by a group of health practitioners from a local tertiary hospital to suit the context of Malaysia. It describes the programme and its effects on mothers' perception, knowledge, perceived barriers and stress, following the programme's implementation in a tertiary NICU in Malaysia. In brief, the study aims to evaluate the Kangaroo Care Intervention effectiveness, through the Maternal Kangaroo Care Education Program (MKC‐EP) on mothers' perceptions, knowledge, perceived barriers and stress in the NICU and 3 months old period of infant post discharge.

## 3 THE STUDY

### 3.1 Design

A quasi‐experimental and longitudinal design study was conducted at the neonatal intensive care unit (NICU) in a tertiary referral teaching hospital.

### 3.2 Methods

The sample size was calculated using the PS‐Power software (version 3) with a two‐sample independent *t*‐test, α = 0.05, effect size 0.22 and power level 0.80 (Faul et al., 2013; Rao et al., 2008). The minimum required sample size was 38 mothers for each arm. An additional 30% (*N* = 12) was added to anticipate non‐adherence. The final sample size calculated was 50 for the control and 50 for the experimental group. The inclusion criteria were: mothers of a first premature infant at postmenstrual age (PMA) between 28 and 36 + 6 weeks of gestation; and mothers who could read and understand English or Malay. The exclusion criteria were: mothers with medical or surgical conditions that require medication that may interfere with breastfeeding; and mothers who had previous premature infants. Mothers who fulfilled the criteria were invited to participate in the study.

Recruitment of participants for the control group was carried out from February 2018 to July 2018, while for the experimental group, from October 2018 to April 2019. A 3‐month hiatus period soon followed to allow for a period of starting afresh where the mothers were educated on the specified program. This way, mothers who were previously recruited during the control period would have been discharged before the commencement of the intensive MKC‐EP. The mothers in the control group received the standard routine care and unit practice. Premature infant being managed in an incubator or under a radiant heater, naked except for a cap and diaper, or wrapped in a cloth. However, the mothers are allowed to touch, hold and feed their premature infants as per standard practice, but the kangaroo care input is not provided after more than 48 hr since hospital admission. After the premature infants were allocated to the standard care (SC) group, they were observed daily and all observations related to the outcomes of the study were documented in the KCPR booklet for the control group i.e. velocity of weight, mode of feeding, type of milk taken and duration of intermittent KC provided (in hours/day/week), date of admission and date of discharge in days.

While after returning home, the mothers recorded all the particulars such as feeding mode, type of milk taken, duration of KC per week and 1 month and 3 months old of the premature infant's weight. Based on the researcher's observation, more than half of the premature infants in the SC group only received 15–30 min of KC from their mothers on an irregular basis in the NICU. The mothers in the SC group visited their premature infants for 30–60 min once a day, for a week before discharge. This was the routine visiting practice of the mothers in the SC group.

The mothers in the experimental group were invited to attend the MKC‐EP conducted by the researchers. The MKC‐EP is a special programme developed to educate mothers with premature infants admitted to the NICU at KC. The MKC‐EP complies with KC guidelines and protocols (USIKC, 2013) and focuses on the psychological and physical aspects relating to the preparation and implementation of KC (before, during and after). It consists of a PowerPoint presentation, a mannequin‐like simulation and a practical session to equip the mothers with knowledge as well as necessary skills to perform KC with their babies. The mothers were guided in performing KC and were provided with a leaflet on KC techniques. The mothers were reminded to perform KC at least 1 hr a day and accumulate to 4–5 hr a week for 3 months. A diary was given to the mothers at the end of the education programme for them to record when KC was performed.

All mothers in the control and experimental groups completed the self‐administered questionnaire soon after consenting to the study's pre‐intervention (T_0_), 1‐month post‐intervention (T_1_) and 3‐month post‐intervention (T_2_). The self‐administered questionnaire contains V parts. Part I consists of 10 characteristic items. Items in Part II, III and IV were adapted from Engler et al. (2002) with slight modifications. Part II contains 20 items to assess the mothers' perceptions of KC. A 5‐point Likert scale was used ranging from strongly disagree (1), disagree (2), neither agree nor disagree (3), agree (4), to strongly agree (5), with a lower mean score reflecting a better perception of KC. Part III consists of 11 items. Respondents were given “true”, ‘false’ and ‘not sure’ to indicate their knowledge of KC, with higher mean scores indicating a better level of knowledge. In Part IV, the respondents were asked to answer 19 items on perceived barriers to practising KC. The scale ranged from very influential (5) to not influential at all (1). A lower mean score of perceived barriers means the mothers are very positive and do not believe that performing KC with their premature infants is an obstacle. Part V is items adapted from Miles et al. (1999), which consists of 7 questions with a 5‐point Likert scale ranging from not at all stressful (1) to extremely stressful (5), to reflect the level of stress faced by mothers with their premature infant's admission into the NICU. The lower the mean stress scores reported by the mothers, the lower the level of stress. Forward translation from English to Malay and backward translation were performed to ensure that the items were of semantic equivalence. The content validity of the questionnaire was ascertained by getting feedback from a panel of experts. The pilot study was conducted with 20 mothers to confirm that the method and instrument used are applicable and feasible. The Cronbach's α for the perception subscale was 0.813, knowledge subscale, 0.785, perceived barriers subscale, 0.873 and for the stress subscale, 0.951, indicating good internal consistency.

### 3.3 Analysis

Data were analysed using SPSS version 25. Descriptive statistics were used to summarize and describe the demographic and social characteristics of the mothers. Chi‐square, general linear model and repeated measures ANOVA were used to analyse data.

### 3.4 Ethics

The study was reviewed and approved by the institutional medical ethics committee (MRECID No: 201765‐5310), registered with the ClinicalTrials.gov (NCT04926402) and performed in accordance with the ethical standards that are outlined in the 2008 Declaration of Helsinki. Permission to use the questionnaire in this study was sought from the original author. The respondents were assured of the confidentiality of this research. Informed written consent was obtained from each respondent after they received a clear and detailed explanation of the nature of the study by the principal researcher. The study complied with the TREND reporting guidelines.

## 4 RESULTS

A total of 100 mother‐infant dyads were recruited and divided into two groups namely the control group (*N* = 50) and the experimental group (*N* = 50). However, only 96 mother‐infant dyads completed the study, with 48 each in the control and experimental groups. In the control group, the reasons for non‐completion included the poor physical health of the baby (*N* = 2). In the experimental group, the reasons for non‐completion included death and the mother's refusal to continue (*N* = 2). The overall mean of the mothers' age was 31.68 years old (*SD* = 4.90). More than half of the mothers (58%, *N* = 56) were between 31 and 41 years old, of Malay ethnicity (54%, *N* = 52), and received up to the secondary level of education (52.1%, *N* = 50). The majority have had a caesarean delivery (87.5%, *N* = 84) with two‐thirds multipara cases (67.7%, *N* = 65). One‐third (37.5%, *N* = 84) of the mothers work in the private sector with a limited maternity leave entitlement of 30 days only. Hence, there is no significant difference in characteristics between the control and experimental groups except for employment status and level of education (*p* < .05) (Table 1).

**TABLE 1:** Characteristics of respondents between control (*N* = 48) and experimental groups (*N* = 48)

Characteristics	Control group *N* = 48(%)	Experimental group *N* = 48(%)	*χ* ^2^	*p*‐value
Age (Mean = 31.68; *SD* = 4.90) years				
20–30	28 (58.3)	12 (25)	5.916^a^	.053
31–41	20 (41.7)	36 (75)		
Race				
Malay	27 (56.3)	25 (52.1)	7.318^a^	.153
Chinese	12 (25.0)	11 (22.9)		
Indian	5 (10.4)	10 (20.8)		
Others	4 (8.3)	2 (4.2)		
Delivery type				
SVD	5 (10.4)	7 (14.6)	0.104^a^	.482
Caesarean section	43 (89.6)	41 (85.4)		
Parity of mother				
Primipara	17 (35.4)	14 (29.2)	0.339^a^	.357
Multipara	31 (64.6)	34 (70.8)		
Highest educational level				
Primary	1 (2.1)	0 (0.0)	14.221^a^	.003*
Secondary	34 (70.8)	14 (29.2)		
Tertiary	13 (27.1)	34 (70.8)		
Employment status				
Government	14 (29.2)	17 (35.4)	9.561^a^	.043*
Private Sector	23 (47.9)	27 (56.3)		
None	11 (22.9)	4 (8.3)		
Maternity leave status				
30 consecutive days	15 (31.3)	21 (43.8)	11.870^a^	.069
60 consecutive days	10 (20.8)	7 (14.6)		
90 consecutive days	12 (25.0)	16 (33.3)		
Not working	11 (22.9)	4 (8.3)		
Post ‐ Partum stress				
Yes	29 (60.4)	32 (66.7)	3.841^a^	.286
No	19 (39.6)	16 (33.3)		
Kangaroo care superficial knowledge				
Yes	13 (27.08)	15 (31.25)	0.995^a^	.005*
No	35 (72.92)	33 (68.75)		

Abbreviation: SVD, Spontaneous Vertex Delivery.

^a^ Chi‐square test between groups.

* Significance at level *p* < .05.

### 4.1 Perceptions of Kangaroo care

A general linear model (GLM) and repeated measure analysis were used to compare the effectiveness of the MKC‐EP on mothers' perception of kangaroo care at T_0_, T_1_ and T_2_. The effect of MKC‐EP between groups was significant (ŋp^2^ = 0.304; *p* < .01). Overall, the mean score for perception towards KC in the control group was significantly different (*p* < .00) with *M* = 3.40 (*SD* = 0.43) compared to *M* = 3.15 (*SD* = 0.24) in the experimental group of mothers after participating in the MKC‐EP. The lower mean scores in the experimental group show that mothers have a positive perception of the KC implementation.

**FIGURE 1:**
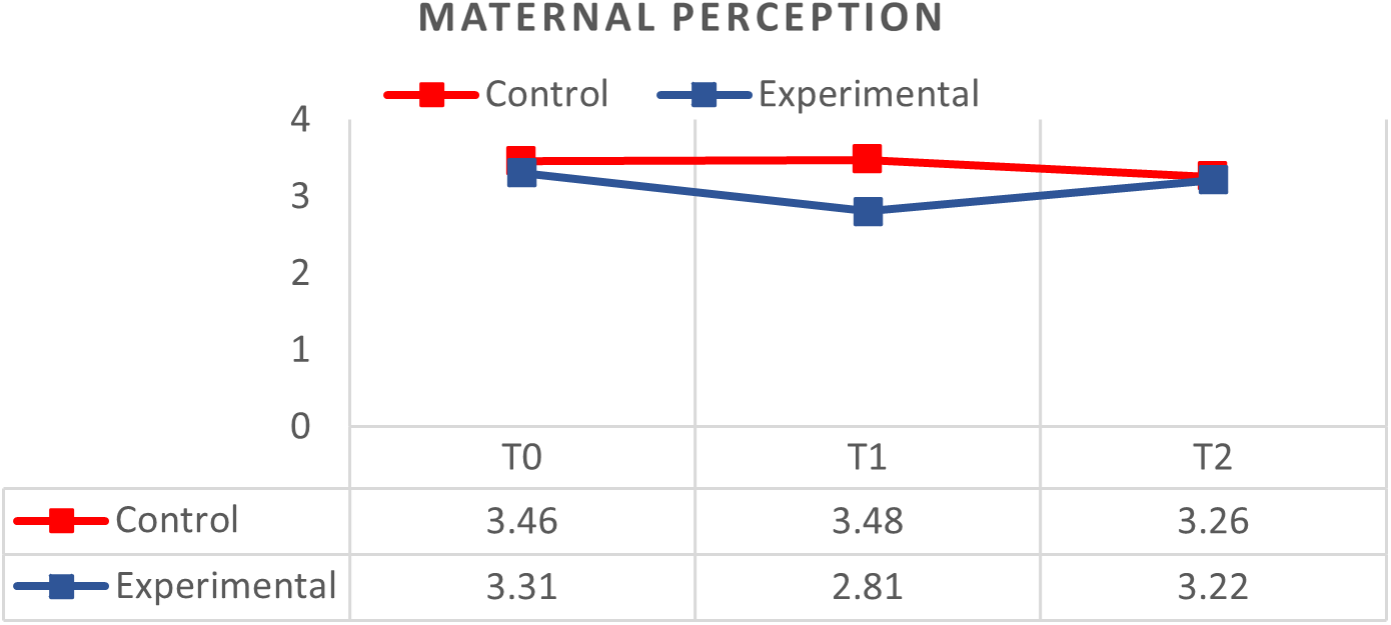
Comparison of respondents between control (*N* = 48) and experimental groups (*N* = 48) on perception of kangaroo care at T0, T1 and T2

In the control group, the mean score of perception of KC was T_0_ (*M* = 3.46, *SD* = 0.04), T_1_ (*M* = 3.48, *SD* = 0.04) and T_2_ (*M* = 3.26, *SD* = 0.05). Results show a significant increase (*p* < .05) in the mean score of perceptions of KC from T_0_ to T_1_ but a decrease in T_2_. As for the experimental group, the reported mean score of perception of KC was T_0_ (*M* = 3.31, *SD* = 0.06), T_1_ (*M* = 2.81; *SD* = 0.04) and T_2_ (*M* = 3.22, *SD* = 0.06). The mean score of the perception of KC significantly (*p* < .00) decreased from T_0_ to T_1_. However, it increased from T_1_–T_2_ (Figure 1). The effect of the MKC‐EP on mothers' perception in the group and across time is small (ŋp^2^ = 0.002; *p* < .05). However, the effect across the group (ŋp^2^ = 0.140; *p* < .00) and between groups (ŋp^2^ = 0.304; *p* < .00) is large.

### 4.2 Knowledge of Kangaroo care

GLM and repeated measures compared the effect of the MKC‐EP on mothers' knowledge related to KC at T_0_, T_1_ and T_2_. The effect of MKC‐EP between groups is large (ŋp^2^ = 0.366; *p* < .00). The mean score of knowledge of KC was significantly different (*p* < .00) between the control group (*M* = 2.16, *SD* = 0.03) and the experimental group (*M* = 2.40, *SD* = 0.03). The higher mean scores in the experimental group show that mothers have higher knowledge of KC implementation.

**FIGURE 2:**
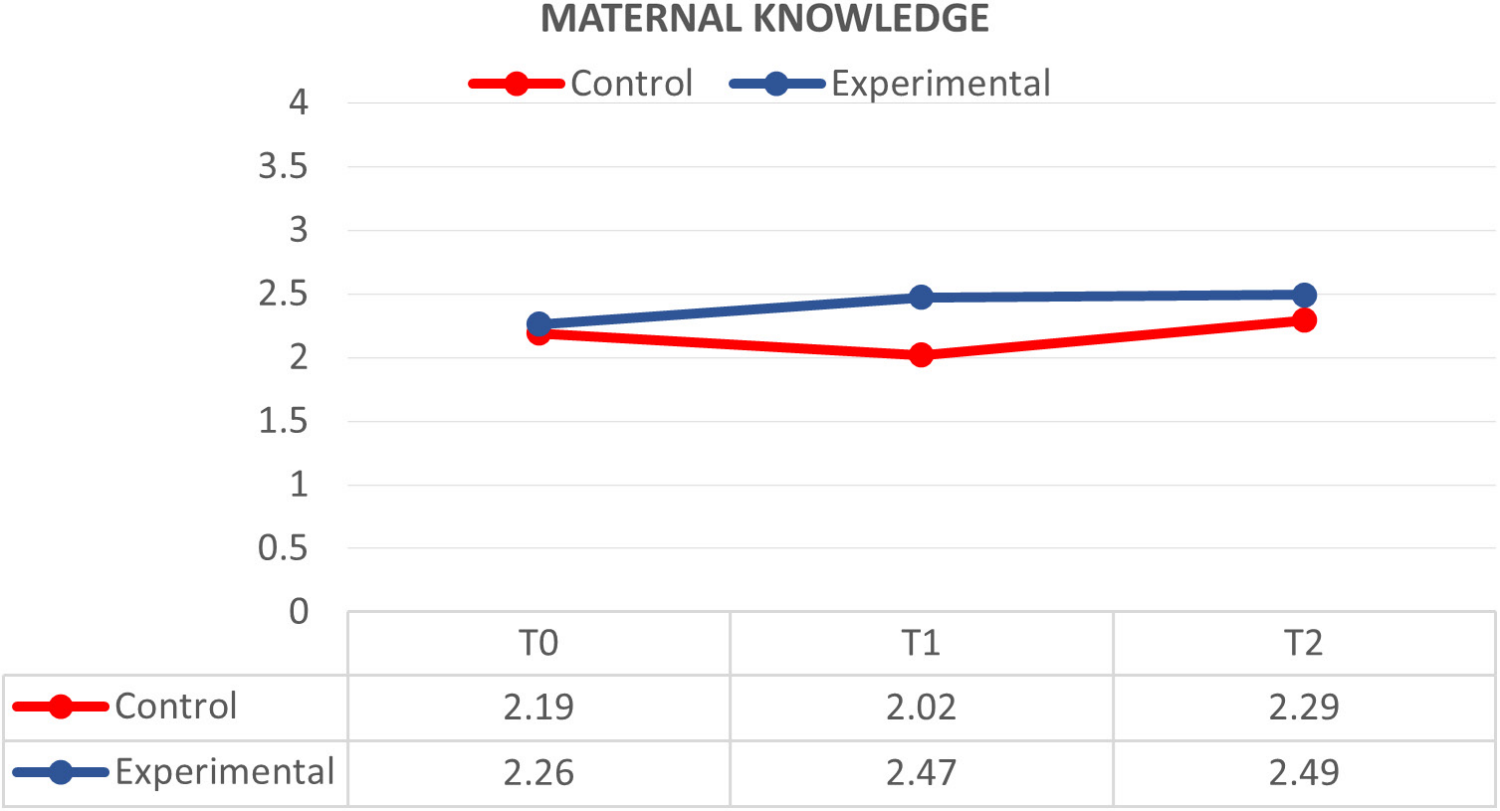
Comparison of respondents between control (*N* = 48) and experimental groups (*N* = 48) on knowledge of kangaroo care at T0, T1 and T2

The control group reported a slightly significant increase (*p* < .05) in the mean score of knowledge of KC from T_0_–T_2_. The mean score of knowledge of KC was T_0_ (*M* = 2.19, *SD* = 0.33), T_1_ (*M* = 2.02, *SD* = 0.33) and T_2_ (*M* = 2.29, *SD* = 0.30). The mean knowledge score of KC was poor from T_0_–T_1_. Nevertheless, there indicated good knowledge from T_1_–T_2_. In the experimental group, the reported mean knowledge score of KC was T_0_ (*M* = 2.26, *SD* = 0.28), T_1_ (*M* = 2.47, *SD* = 0.11) and T_2_ (*M* = 2.49, *SD* = 0.19). The mean score of knowledge among mothers who participated in the MKC‐EP significantly improved (*p* < .00) (Figure 2). The effect of the MKC‐EP on mothers' knowledge in the group and across time is small (ŋp^2^ = 0.099; *p* < .01). However, the effect across the group (ŋp^2^ = 0.100; *p* < .01) and between groups (ŋp^2^ = 0.366; *p* < .01) is large.

### 4.3 Perceived barriers to Kangaroo care

General linear model and repeated measures were used to compare the effect of the MKC‐EP on mothers' perceived barriers towards KC at T_0_, T_1_ and T_2_. The effect is medium between groups (ŋp^2^ = 0.083; *p* < .01). There was a significant difference (*p* < .01) in mothers' perceived barriers towards KC between the control group, *M* = 2.57 (*SD* = 0.08) and the experimental group after participating in the MKC‐EP, *M* = 2.20 (*SD* = 0.09).

**FIGURE 3:**
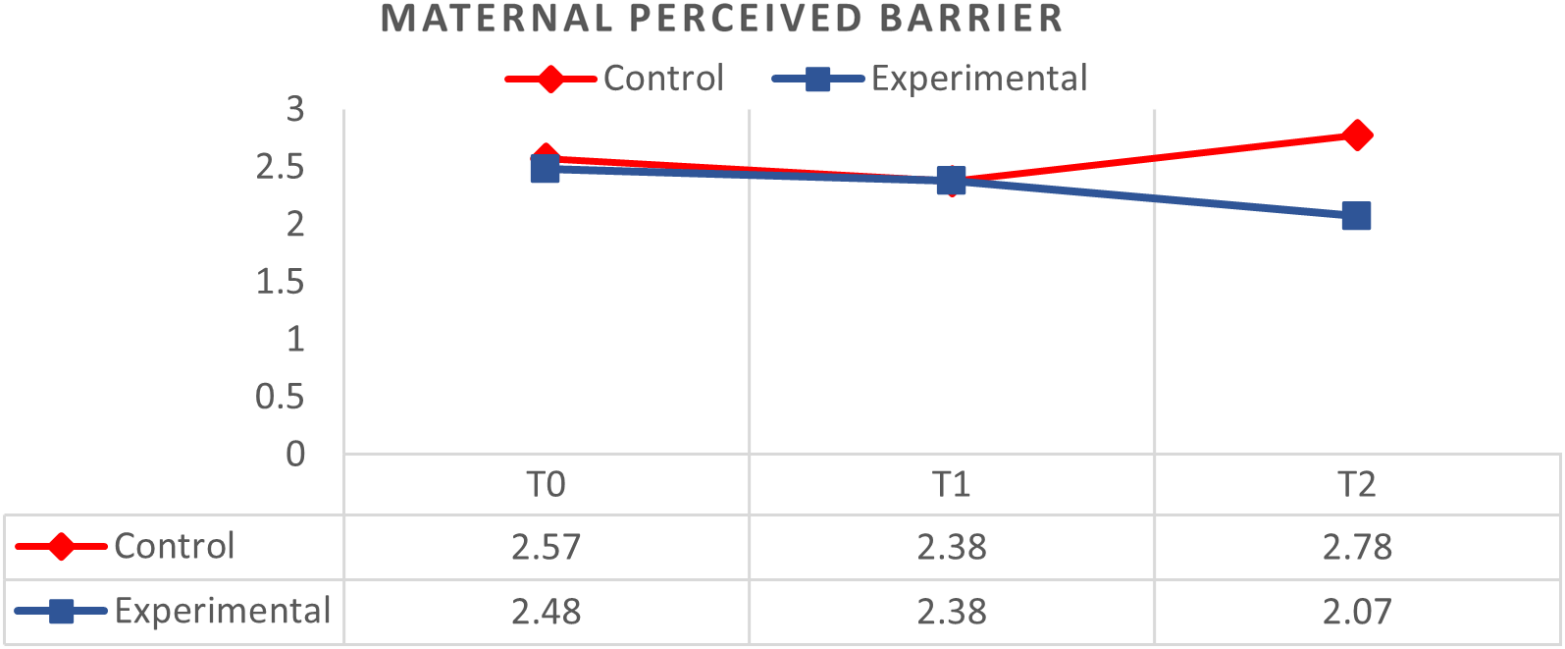
Comparison of respondents between control (*N* = 48) and experimental groups (*N* = 48) on perceived barrier of kangaroo care at T0, T1 and T2

The control group reported an overall significant increase (*p* > .05) in the mean score of perceived barriers towards KC from T_0_–T_2_. The mean score of perceived barriers towards KC was T_0_ (*M* = 2.57, *SD* = 0.12), T_1_ (*M* = 2.38, *SD* = 0.14) and T_2_ (*M* = 2.78, *SD* = 0.08). However, only T_1_ showed a slight decrease in the score of perceived barriers towards KC, which was close to the total mean score in the control group. As for the experimental group, the mean score of perceived barriers towards KC was T_0_ (*M* = 2.48, *SD* = 0.12), T_1_ (*M* = 2.38, *SD* = 0.14) and T_2_ (*M* = 2.07, *SD* = 0.09). The mean score of perceived barriers towards KC decreased from T_0_–T_2_ in the experimental group (Figure 3). The MKC‐EP on mothers' perceived barriers has a small effect across time (ŋp^2^ = 0.006; *p* < .05). However, there is a medium effect between groups and across time (ŋp^2^ = 0.069; *p* < .01) and within groups (ŋp^2^ = 0.083; *p* < .01).

### 4.4 Mothers' stress related to premature infant's admission to NICU

A general linear model (GLM) analysis was conducted to study the stress level among mothers with premature infants admitted to the NICU, compared with the effect of the MKC‐EP on the mothers' stress levels at T_0_, T_1_ and T_2_ between the control and experimental groups. Results show that with the implementation of MKC‐EP, the effect is large between the control and experimental groups (ŋp^2^ = 0.345; *p* < .01). Overall, the mean score of stress was higher in the control group (*M* = 3.74, *SD* = 0.09) compared with the experimental group (*M* = 2.96, *SD* = 0.09) (*p* < .001).

Mothers in the control group did not report a significant difference (*p* > .05) in the mean score of stress related to premature infants admitted to the NICU from T_0_–T_2_. The mean score of stress was (*M* = 3.69, *SD* = 0.14) at T_0_, (*M* = 3.73, *SD* = 0.20) at T_1_, and (*M* = 3.80, *SD* = 0.07) at T_2._ Mothers in the control group with premature infants admitted to the NICU reported an increase in stress from T_1_–T_2_.

**FIGURE 4:**
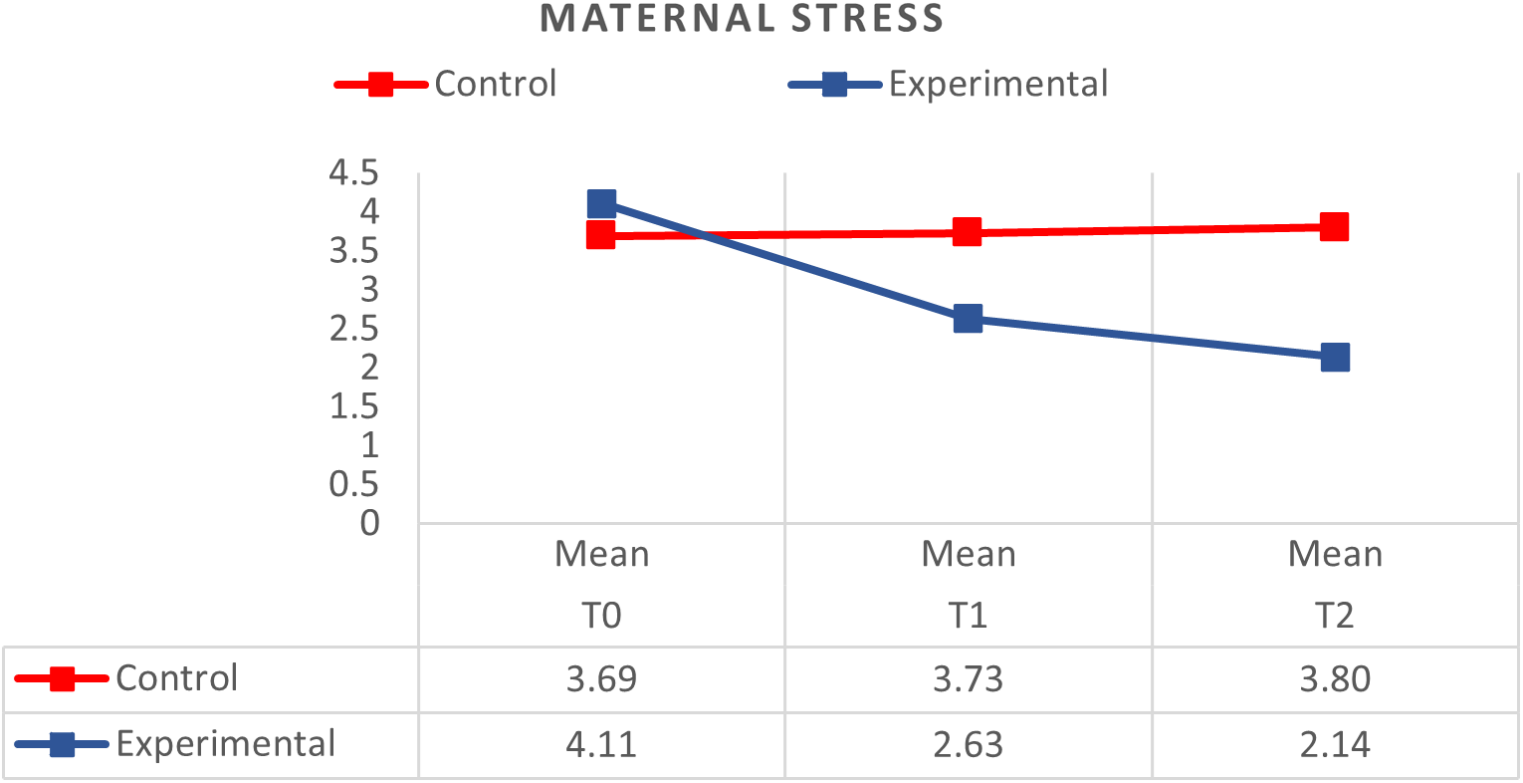
Comparison of respondents between control (*N* = 48) and experimental groups (*N* = 48) on stress related to premature infant admitted to NICU in the initiation of kangaroo care at T0, T1 and T2

In the experimental group, the mean score of stress related to premature infants admitted to the NICU was T_0_ (*M* = 4.11, *SD* = 0.15), T_1_ (*M* = 2.63, *SD* = 0.10) and T_2_ (*M* = 2.14, *SD* = 0.07), which indicates a decrease at T_2_ in comparison to T_0_. The mean level of stress was significantly reduced (*p* < .00) from T_0_ to T_2_ after the participants' involvement in the structured MKC‐EP in the experimental group (Figure 4). There is a large effect on maternal stress level across time (ŋp^2^ = 0.165; *p* < .01), between time and groups (ŋp^2^ = 0.141; *p* < .01), and between groups (ŋp^2^ = 0.345; *p* < .01).

## 5 DISCUSSION

### 5.1 Mothers' perception of Kangaroo care

It is noteworthy that the MKC‐EP has brought a significant change in the mothers' perception of KC's advantages for their premature infants. Thus, more than half of the mothers in the experimental group had a good perception towards implementing KC until their premature infants reached 3 months old. MKC‐EP has positively increased the perception of KC among mothers with premature infants in the NICU.

This finding is supported by previous research conducted by Nirmala et al. (2006) to assess KC perceptions among mothers and health care workers. Their study highlighted that all mothers said that KC had improved bonding and made them feel good, fulfilled and pleased to contribute to the care of their infants. On the contrary, Verklan and Walden, (2015) shared different outcomes. In their longitudinal study in Ghana, the mothers feared that the practice of KC would not be acceptable in the community as babies are traditionally carried on the mother's back instead of on their chests (Verklan & Walden, 2015). In this case, conducting the MKC‐EP can effectively improve the perception of KC and bonding between mothers and premature infants.

Overall, it is proven that the MKC‐EP can change the perception of KC among mothers about its implementation and quality of premature infant care in situations when their infants are admitted to the NICU. Thus, conducting the MKC‐EP can improve the perception of KC among mothers of preterm infants in the NICU. In the future, KC should be provided for preterm infant care as a standard practice and evidence‐based nursing intervention approach.

### 5.2 Mothers' knowledge of Kangaroo care

The results of this study show that mothers predominantly had inadequate knowledge about KC before the intervention program. With adequate exposure and practice, they have acquired a better understanding of the KC technique and improved its implementation with their premature infant. It is evident that the educational intervention program, which includes hands‐on instruction, was effective in increasing KC awareness among mothers and was successful in enhancing their knowledge of KC. This study is similar to previous studies, particularly in conducting the MKC‐EP for mothers to develop their KC knowledge. Thus, these findings are consistent with other research that shows positive results in the implementation of an educational approach that can improve maternal knowledge of KC. Case in point, the pre‐experimental one‐group pre‐test study conducted in Gujarat by Gayathri et al. (2016) reported a notable increase in the overall knowledge score for the post‐test compared with the pre‐test, indicating the effectiveness of the structured teaching program. The study also concluded that the structured teaching programme was an effective method to teach and enhance knowledge of KC among mothers of infants with low birthweight. However, the study evaluated effectiveness just after 7 days of delivery. In contrast, the researchers in this study took the opportunity to evaluate the mothers' knowledge at 3 points in time, starting from 48 hr after delivery (28 weeks), after 1 month and after 3 months. Comparing the three phases is necessary as it would help the researchers monitoring the consistency of knowledge that the mothers have received accordingly. Despite the increase in knowledge in the experimental group of mothers, their moderate levels of knowledge suggest that there exist gaps in the knowledge and misunderstandings about KC that could be improved, possibly even after the intervention.

Another study conducted by Panchal and Ravindra (2016) also supports the application of a structured intervention programme. They have found that a planned teaching program in KC is a successful method of educating post‐natal mothers. A mother's knowledge of KC is crucial to successfully implementing KC during her baby's admission to the NICU (Ong et al., 2019). This implies that management and nurses need to step up their efforts and introduce more initiatives such as ongoing education on KC awareness and on the benefits of breastfeeding in the NICU setting in the future.

In short, the results of this study indicate that mothers from all over the world need a continuous structured education delivered via flip charts, videos and informative leaflets on the evidence‐based practice and benefits of KC to overcome their lack of knowledge of KC.

### 5.3 Mothers' perceived barriers to Kangaroo care

Although KC is a key intervention package in preterm infant health initiatives, there is limited systemic information available on the barriers that mothers and other stakeholders face in KC practice (Seidman et al., 2015). In resource‐rich countries, KC is seen as complementary to incubator care, so continuous KC is rare. KC implementation in hospitals is motivated mainly by a desire to humanize a medical experience and to partially fulfil the requirements set out in the Baby‐Friendly Hospital Initiative (BFHI) (Britney & Sonia, 2014; UN Children's Fund, 2018).

The current study shows that there are statistically significant differences in the proportions of reluctance and compliance concerning the implementation of KC between the control and experimental groups. The perceived barriers in this study refer to measures that the mothers considered influential to them performing KC. It is noted that although the perceived barriers were significantly reduced for the control group compared to the experimental group, one‐third of the mothers did not have the confidence to perform KC consistently in the NICU and after returning home.

Mothers in this study understood the benefits of KC, but their attitude and awareness might have led them not to continue the practice consistently. Further, as the majority of the participants are Malay and Muslim, the most predominant obstacle to KC in terms of maternal factors is the reluctance and discomfort of the mother in exposing her breasts during the KC session. This is similar to the findings of studies about initiated barriers to performing KC among mothers through nurses' evaluation. Mahboobeh et al. (2016) reported that 60% of nurses in their study believed that the key barrier to KC was the lack of continuous attendance of mothers in the ward. Other mother‐related barriers were the mother's fear of touching her premature infant, the mother's fear of ward equipment and twin or triplet infants.

The mothers in the experimental group seemed to have developed self‐confidence by touching, handling, changing diapers, feeding and performing KC with their premature infants. This indicates that the MKC‐EP has significantly affected the mothers in the experimental group and built their self‐confidence in implementing KC. A systematic review conducted by Seidman et al., (2015) revealed that the main obstacles to practising KC are related to resources which include “facility environment/resource issues”, “negative perceptions of staff behaviour or communication between staff”, “lack of assistance with KC procedures” and “weak KC/infant health awareness”. The successful implementation of MKC‐EP reduces mothers' perceived barriers towards performing KC with their premature infants.

### 5.4 Mothers' stress related to premature infant's admission to NICU

Mothers facing maternal stress find it difficult to cope or deal with the challenging experience resulting from their premature infant being admitted to the NICU. In this study, mothers in the experimental group reported significantly less stress after performing KC at each phase of the MKC‐EP. These results are believed to have occurred because mothers in the experimental group were able to approach the NICU equipment, get used to the sensations of the NICU and were able to touch and hold their infant as they performed KC on a daily basis. The mothers may have developed maternal roles by engaging in the care of their infants. The findings are consistent with earlier studies that reported psychological stress as one of the factors that influence a mother's potential to interact effectively with her premature infant and that stress sensation was found to be related to less warm and less responsive maternal behaviour (Assel et al., 2002; Ong et al., 2019; Feeley et al., 2011; Zelkowitz et al., 2009). Therefore, an emotionally charged event like this is when both mother and infant are willing to adopt new behaviours to minimize health risks. Therefore, performing maternal roles can reduce post‐partum stress. Apart from that, the other benefits shown for premature infants in other clinical trials, especially randomized controlled trials that have been conducted by other researchers, show the appropriateness of the use of this intervention in the NICU for premature infants. A related study of Family Fostering Intervention (FNI) by (Hane et al., 2015; Welch et al., 2013; Welch et al., 2015 & Welch et al., 2016) demonstrates the benefit of maternal emotional expression with premature infant‐directed speech during skin‐to‐skin contact that will be benefited by wraparound NICU support and education. The MKC‐EP significantly increases parenting abilities and reduces stress levels in mother‐infant dyads.

## 6 LIMITATIONS

Although the study has overcome key methodological limitations of previous studies, it still has several limitations. First, we were only able to compare the sample in this study with non‐randomized samples even when the two groups of mothers matched in characteristics. The study included only singleton births, although a high percentage of preterm infants born in Malaysia are twins or triplets. Second, the inclusion of multiple births would have confounded the relationship between prematurity and the outcome variables as it would have been difficult to find a sufficient number of preterm multiple births, making it impossible to control for differences in the two groups. Third, there may be seasonal effects, individual changes between the control and experimental groups, or other variables that skew data because the control and experimental groups were not run at the same time. Fourth, this study was only recently posted on CT.gov shortly after it was completed. Fifth, our analysis has only differentiated the effect of MKC‐EP on the mothers performing KC before and after the intervention. Sixth, we acknowledge that KC intensity is only one measure over 3 months of infant corrected age. The study should have measured until 6 months for exclusive breastfeeding status. Further studies should involve parents with various levels of perception, knowledge, perceived barriers and stress, which would make for a more informative analysis and possibly produce quality outcomes concerning the potential and relative effect of KC on premature infants.

## 7 CONCLUSION

Kangaroo care in the neonatal environment is greatly inhibited hampered by a lack of formal training and education. If kangaroo care is offered, it is based on the mother's ability and confidence in its effectiveness. The results of this study showed that MKC‐EP improved the quality of maternal caregiving behaviours on perception, knowledge, perceived barriers and stress during the time the infant spent in the neonatal intensive care unit (NICU) and 3 months old after being discharged. MKC‐EP also could be a viable and practical strategy to reduce the impact of premature birth on maternal stress and anxiety symptoms in clinical and at‐home contexts. Despite this, MKC‐EP includes some activities that are part of other nurture‐based interventions, such as kangaroo care performance, infant touch, breastfeeding and weight gain. This study supports including these activities in a routine NICU care programme that focuses on mother‐infant bonding.

## AUTHOR CONTRIBUTIONS

All authors contributed equally to this work.

All authors have agreed on the final version and meet at least one of the following criteria [recommended bythe ICMJE (http://www.icmje.org/recommendations/)]:

• substantial contributions to conception and design, acquisition of data or analysis and interpretation of data;

• drafting the article or revising it critically for important intellectual content.

## FUNDING INFORMATION

This research is not funded by any specific grant from any funding agency in the public, commercial, or not‐for‐profit sectors.

## CONFLICT OF INTEREST

The authors declared no conflict of interest in relation to this study.

## ETHICAL STATEMENT

The study complied with the principles of the Declaration of Helsinki.

## Data Availability

The data that support the findings of this study are available from the corresponding author upon reasonable request.
